# Intracranial pressure and hypothermia

**DOI:** 10.1186/cc11281

**Published:** 2012-06-07

**Authors:** Claudius Thomé

**Affiliations:** 1 Department of Neurosurgery, Innsbruck Medical University, Innsbruck, Austria

## 

Although the neuroprotective potential of hypothermia is well known and has been established experimentally, its clinical use is limited to selected indications [1], as large trials have yielded disappointing results [2]. This has been mainly attributed to the side effects of hypothermia in critically ill patients and problems with rewarming.

Intracranial hypertension is a major problem in neurocritical care and particularly in patients with subarachnoid hemorrhage (SAH) and traumatic brain injury (TBI), causing death if uncontrolled. As trials on prophylactic hypothermia, for example, for TBI have not been successful in improving outcome, its routine use can currently not be recommended. However, there are many literature reports demonstrating enormous efficacy of hypothermia to reduce elevated intracranial pressure (ICP). Mechanisms of action are thought to be the reduction of metabolism and perfusion and the reduction of edema besides others. Studies have indicated that therapeutic efficacy is sufficient for ICP control at mild hypothermia of 35°C, thus minimizing detrimental effects. In desperate clinical situations hypothermia is used to control intracranial hypertension both for TBI and SAH, but it has recently been applied only as a last resort. Other second-tier therapies and surgical maneuvers like decompressive craniectomy have been popularized instead, although their efficacy is still questioned as well. The knowledge and experience with therapeutic hypothermia has advanced in recent years and the problems of side effects and most importantly rewarming can be better addressed [3]. The latter has been a tremendous problem in patients with uncontrollable ICP, as despite its initial efficacy ICP problems recurred, if hypothermia was stopped prematurely. This goes in line with a recent metaanalysis that stressed the importance of prolonged hypothermia (48 hours to 5 days) and of slow rewarming (<1°C/4 hours). As a consequence the Eurotherm3235Trial was initiated to investigate the effect of hypothermia particularly for intracranial pressure reduction [4]. It has to be awaited whether this will foster the use of hypothermia to treat elevated ICP or whether we will stick with the policy of controlled normothermia.
